# Economic Burden of Contraception Management in Spain

**DOI:** 10.36469/9785

**Published:** 2018-04-16

**Authors:** Inmaculada Parra Ribes, Juan José Rascón Poza, Ezequiel Pérez Campos, Ignacio Bugella Yudice, Maria Jesús Rodríguez Domingo

**Affiliations:** 1 Unidad de Salud Sexual y Reproductiva de Sueca, Valencia, Spain; 2 Centro de Salud Mediterráneo, Almería, Spain; 3 Hospital de Requena, Valencia, Spain; 4 Centro Planificación de Getafe, Madrid, Spain; 5 Centro ASSIR Viladecans, Barcelona, Spain

**Keywords:** economic burden, intrauterine system, long-acting reversible contraceptives, reversible contraception, short-acting reversible contraceptives, unintended pregnancy

## Abstract

**Background:**

A wide variety of contraceptive methods are available, some of them reimbursed by the Spanish National Health System (SNHS). However, the number of unintended pregnancies (UP) is still significant, leading to a high economic burden, mainly derived from non-adherence to and the incorrect use of contraceptive methods.

**Objectives:**

This study aims to estimate the economic burden associated with reversible contraception management in Spain, from the perspectives of both the SNHS and women, over a 5-year period.

**Methods:**

A survey was performed to identify contraception management in Spain based on the experience of a panel of six expert gynecologists. An economic model was conducted to quantify the current burden of contraception according to healthcare resources use over 5 years. The costs included in the analysis were diagnostic tests, initial and follow-up consultations, methods acquisition costs, and UP derived from therapy failure.

**Results:**

Reversible contraception costs in Spain amount to €12.5 billion over a 5–year period. Condoms and combined oral contraceptives (COC) account for 86.8% of the total cost and the other methods only 13.2%. There are differences in contraceptive use according to women’s age. Short-acting reversible contraceptives (SARC) such as COC, condom and vaginal ring, are most commonly used by younger women. However, SARC are correlated with the highest failure rate, resulting in over €7.2 billion cost, explained by the high number of UP. Long-acting reversible contraceptives (LARC), such as the levonorgestrel-releasing intrauterine system (LNG-IUS20), implant and copper intrauterine devices (IUD), are selected by women over 35 years of age due to user-independent compliance. SARC methods result in a higher cost per woman over 5 years: vaginal ring €2427.8, patch €2402.6, condom €2060.1 and COC €1895.1; while LARC methods are the most economic option per women: LNG-IUS20 €630.4, copper IUD €658.2, LNG-IUS12 €703.8, intramuscular injectable €907.8 and implant €940.5.

**Conclusions:**

LARC methods result in lower costs compared with SARC options from the perspectives of the SNHS and women, explained by user-independent compliance, preventing a significant number of UP and its elevated economic burden. An increased LARC methods use could avoid UP events, leading to significant cost-savings for the SNHS.

## Background

A wide variety of contraceptive products are available in Spain and some, such as oral contraceptive (OC) pills, injections, and implants, are reimbursed by the Spanish National Health System (SNHS). However, the number of unintended pregnancies (UPs) is still considerable, leading to high potentially avoidable expenditures by the SNHS with greater use of user-independent compliance methods, such as long-acting reversible contraceptives (LARC).^1^

From 1997 to 2007, Spain experienced a 30.0% increase in the use of contraception.^2^ In the Spanish contraception survey of 2016, 71.1% of women of childbearing age, between 14 and 49 years, responded that they used contraceptive methods, while 4.1% did not know/did not answer, and 24.7% of women did not use any contraception.^3^ Among the latter, 8.3% did not have sexual intercourse, but the remaining 16.4% of women may be at risk of UP due to the lack of contraception use, or even the use of ineffective methods (1.0%), such as coitus interruptus or natural methods.^3^ Among all contraceptive methods used in Spain, reversible methods are employed by 87.3% of women, that is to say, by up to 6.8 million women.^4^ Reversible methods include short-acting reversible contraceptives (SARC) (combined oral contraceptives [COC], gestagen-only oral contraceptives [OCs], transdermal patch, vaginal ring, male condom) and LARC (subdermal implant [Implanon NXT®, progestagen-only implant], intramuscular injectable [Depo-Provera®, Medroxyprogesterone Acetate Injectable Suspension)], LNG-IUS and copper intrauterine device [IUD]. The most common methods are male condom and COC, used by 45.7% and 34.9% of women, respectively.^3^

Despite an increase in the use of contraception in recent years, the number of elective abortions has risen, mainly attributed to the lack of compliance with or incorrect use of methods requiring frequent dosing.^2^ A recent study among Spanish women who had had an induced abortion showed that 64.0% of them were using contraceptive methods, mainly condom (40.0%) and COC (14.0%).^5^ Failure of the method due to incorrect use was reported by 77.0% of the women using condoms and by 84.0% of those using hormonal contraception.^5^

A better and deeper understanding of the country’s real situation concerning contraception is required to define and implement appropriate sexual health and contraception policies.

Currently, there are no data on the economic burden of contraception management in Spain for the National Health System. This study is the first to describe the current situation of reversible contraception in Spain and estimate its economic burden from the perspective of both users and the SNHS over a 5–year period.

## Methods

In 2015, an ad-hoc questionnaire was designed to ascertain the current utilization of healthcare resources associated with reversible contraception management from the perspective of the SNHS. The questionnaire was structured into five main sections: current use of contraceptive methods in Spain, method withdrawal, medical consultations and diagnostic tests, methods of preference according to women’s age, and prescription patterns/contraception recommendations. Separate estimates were performed in 5-year age groups in order to explore age-specific trends. The questionnaire was answered by a panel of six gynecologists who are experts on contraception.

An economic model was built to estimate the total cost of reversible contraception management in Spain over 5 years based on the inputs provided by the experts. The model represents a 5-year period of use, which allows comparison between different reversible contraception methods with different efficacy duration, ie, SARC and LARC. The population included in the analysis was women of childbearing age, between 14 and 49 years, managed by the SNHS.

The model includes the most representative healthcare costs associated with the current contraceptive care pathway in Spain: 1) cost of the first visits to the gynecologist to initiate contraceptive therapy; 2) cost per patient and contraception method of diagnostic tests not included in routine consultation; 3) cost per patient and contraception method of the number of follow-up consultations during the 5-year period; 4) contraception acquisition cost during a period of 5 years, either reimbursed by the SNHS or paid by the user, and 5) cost of therapy failure resulting in a UP over 5 years. Costs are expressed in euro (€) 2017. Other costs, such as adverse events related with each contraceptive option (i.e. bleeding irregularities, uterine perforation, pelvic infection or ovarian cysts) were not included in the economic analysis.

The use of reversible contraception was obtained from a survey conducted by the Spanish Society of Contraception (SEC).^3^ However, the LNG-IUS12 contraceptive method was not included in this survey since it was not available on the market then. Thus, LNG-IUS12 utilization was provided by the experts based on their experience in the routine clinical practice.

### Cost of Contraceptive Therapy Initiation

Contraceptive therapy initiation consists of the first consultation with the gynecologist and diagnostic tests (ultrasound and blood tests), frequently carried out prior to initiating therapy. Unitary costs were calculated according to the average price of the catalogues of public prices of healthcare services of the main Spanish Autonomous Communities (Andalusia,^6^ Catalonia,^7^ and Madrid^8^), weighted by population, and updated to 2017, based on the Consumer Price Index.^9^ The unit cost of the first gynecologist consultation in Spain is €79.1, while the unit cost for diagnostic tests is €23.5 for ultrasounds and €29.9 for blood tests. For the diagnostic tests, costs were weighted by frequency of use. Total contraceptive therapy initiation cost per method was calculated considering the number of women using each method and the annual frequency of diagnostics and initial gynecologist consultations (Table 1, Table 2).

### Cost of Follow-up Consultations

The number of follow-up consultations to the gynecologist varies according to the contraceptive method chosen. Total follow-up costs were estimated based on the number of women using each method, the average number of subsequent consultations per year according to the data provided by the experts, and the unit cost of the follow-up visit, for a 5-year period. The unit cost of a follow-up visit is €52.4, calculated as for the initial visit^6^–^8^ (Table 3). The total cost associated to medical management comprises the initial visit, the follow-up consultations and diagnostic tests (Table 4).

### Cost of Contraceptive Method Acquisition

The cost of the acquisition of each contraceptive method (retail price) was obtained from the records of the SEC^3^ and the database of the General Council of Official Colleges of Pharmacists.^10^ A price reduction was applied in those methods currently reimbursed by the SNHS according to Royal Decree 8/2010.^11^ The cost associated with each reversible contraceptive method during a period of 5 years was estimated based on the number of users of each method, the frequency of use and the method acquisition price (Table 5).

### Contraceptive Therapy Failure/Unintended Pregnancy

The cost of UP was included in the economic burden analysis, considering a time period of 5 years. Each contraceptive method is associated with a percentage of UP (Table 6) attributable to an imperfect method of use (treatment failure rate) in routine practice.^1^,^12^ This frequency of UP was used to estimate the number of failures per method in a period of 5 years. Each failure leads to a UP, with four possible outcomes: live birth, induced abortion, fetal loss or ectopic pregnancy. The annual number of these events was obtained from the Spanish Ministry of Health, Social Services and Equality (MHSSE)^13^ and the Spanish National Statistics Institute^4^ consistent with the methodology used in a previous recent publication.^1^ The frequency of live births was 50.1%, induced abortion 43.3%, ectopic pregnancy 6.5%, and fetal loss 0.2%.^4^,^13^ The cost per UP outcome was obtained from the Diagnosis Related Group codes published by the MHSSE.^13^ The unit cost associated to each of UP outcome is €2027.9 (live birth), €1487.3 (induced abortion), €1487.3 (fetal loss) and €1991.3 (ectopic pregnancy).^14^

Finally, the cost of UP was the weighted average cost of the four possible UP outcomes. The average cost per UP in Spain is estimated at €1790.9.

### Contraception Cost per Woman and Method

Finally, the average cost per woman and method, considering total 5-year costs of gynecologist consultations and diagnostic tests, acquisition cost and the cost of UP derived from therapy failure was estimated and compared.

## Results

In Spain, 87.3% of women use reversible contraceptive methods based on the SEC survey,^3^ resulting in approximately 6.8 million users (Table 1). The usage of LNG-IUS12 was estimated based on the opinions of experts, who considered that 5% of LNG-IUS20 users and 2.5% of copper IUD users switched to LNG-IUS12. As a result, LNG-IUS12 is used by 0.4% of women, LNG-IUS20 by 5.2% and copper IUD by 5.5%. The most commonly used reversible methods are male condom (45.7%) and COC (34.9%).

The preferred contraceptive methods according to women’s age are COC, male condom and vaginal ring for women between 15 and 29 years of age, according to data provided by experts based on their experience. In women between 30 and 34 years of age, preferences are for COC, male condom and LNG-IUS20, while from 35 to 49 years, LNG-IUS20 is the preferred contraceptive method, followed by male condom and copper IUD. Women over 35 years of age prefer long-term contraception methods, such as LNG-IUS and copper IUD, due to safety and user non-dependence compliance. Moreover, when contraception is considered at mid-term based on input obtained from the experts, women’s preferences tend towards greater use of long-term methods: LNG-IUS20 and copper IUD are each used by 11.0% of women and the vaginal ring by 10.0%, while male condom and COC are used by only 23.0% and 29.0% of women, respectively.

On average, the percentage change in contraception method is 68.0% for injection, 60.0% for male condom, 53.0% for gestagen-only OCs, 40.0% for patch, 37.0% for COC, 33.0% for vaginal ring, 32.0% for implant, 25.0% for copper IUD, 10.0% for LNG-IUS20 and 9.0% for LNG-IUS12. This data was provided by experts. The main reason for method withdrawal is the onset of adverse events (AE), followed by discomfort and lack of compliance. Considering each reversible method, the main reasons for withdrawal from COC is lack of compliance, followed by AE and discomfort. Women using gestagen-only OCs change mainly because of AE, lack of compliance and discomfort, while the patch is normally replaced by another method due to AE, discomfort and misuse. Vaginal ring, LNG-IUS and copper IUD users typically decide to change their contraceptive method mainly due to AE, other reasons (amenorrhea, overweight) and discomfort. Male condom withdrawal responds to the lack of compliance, misuse and discomfort, while implant and injection is mostly withdrawn because of AE.

### Cost of Contraceptive Therapy Initiation

Table 1 shows the total costs of the first consultation with the gynecologist to initiate contraceptive therapy. Total costs of diagnostic tests required to initiate contraceptive therapy with each method are shown in Table 2. The highest costs come from the COC method, since it is the one that requires the most diagnostic tests, followed by copper IUD, LNG-IUS20, and vaginal ring.

### Cost of Follow-up Consultations

The methods requiring the highest number of follow-up visits are implant and copper IUD to monitor bleeding adverse events. However, the highest cost is accounted for by COC and male condom, the most frequently used methods in Spain (Table 3).

### Total Cost of Medical Management

The total 5-year cost of medical care (initial and follow-up consultations) and the cost of the diagnostic tests amounts to €2.1 billion (Table 4). The main cost drivers are COC (€850.3 million) and male condom (€629.3 million).

### Contraceptive Method Acquisition Costs

The acquisition cost for each contraceptive option during a period of 5 years is shown in Table 5. LARC methods (LNG-IUS20, LNG-IUS12, copper IUD, implant and injection) are associated with the lowest cost, while the highest cost corresponds to SARC methods, mainly COC, male condom, and vaginal ring.

### Cost of Unintended Pregnancies

The annual direct cost of UP in a period of 5 years is €7.3 billion. Condoms and COC account for €5 billion (68.3%) and €1.9 billion (26.1%), respectively, while all LARC (LNG-IUS20, LNG-IUS12, copper IUD, implant and injectable) only amount to €45.5 million (0.6%). The lowest costs of UP due to method failure come from implant, LNG-IUS12 and LNG-IUS20, representing only 0.1% of the total UP cost (€4.5, €17.9 and €17.9 per woman over 5 years, respectively). The average cost per woman of UP in 5 years is estimated at €1078.6 (Table 6).

### Total Costs of Reversible Contraception

The total 5-year costs of the management of reversible contraception in Spain amount to €12.5 billion, mainly coming from male condom (51.0%) and COC methods (35.8%) due to higher frequency of use and a higher number of UP. These two methods are associated with a high rate of failure (18.0% and 9.0%, respectively) (1,12). The other methods, representing 13.2% of total costs, are: vaginal ring (7.2%), patch (1.0%), copper IUD (2.0%), LNG-IUS20 (1.8%), implant (0.7%), gestagen-only OCs (0.3%), LNG-IUS12 (0.2%) and injection (0.2%) (Table 7).

**Figure 1:**
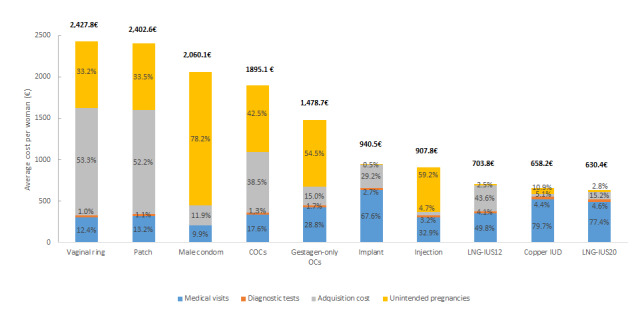
Average Cost per Woman and Contraceptive Method in a Period of 5 Years For each contraceptive method, cost components are expressed as percentages and the average 5-year cost per woman and method is shown in euro. COC: Combined Oral Contraceptives; OCs: Oral Contraceptives; IUD: Intrauterine Device; LNG-IUS12: Levonorgestrel-releasing intrauterine system Jaydess 12μg/day; LNG-IUS20: Levonorgestrel-releasing intrauterine system Mirena 20μg/day

In the analysis of 5-year costs per woman and method, the methods with the highest costs are vaginal ring (€2427.8) and patch (€2402.6), while the least costly options are LNG-IUS20 (€630.4), copper IUD (€658.2) and LNG-IUS12 (€703.8) (Figure 1).

## Discussion

The contraceptive use patterns among Spanish women are influenced by factors such as age, number of children, socio-economic status, and information received during contraceptive counselling.^15^ Despite the wide availability of contraceptive methods in Spain, UP are still numerous, resulting in elevated costs for the SNHS.^1^ However, the economic burden of contraception management in Spain had not been previously assessed.

This study is the first to estimate the total cost of reversible contraception management in Spain over a period of 5 years from the perspective of the user and the SNHS, considering the main healthcare resources used, ie, first visit and diagnostic tests to initiate contraceptive therapy and follow-up consultations, contraceptive method acquisition cost, and related UP costs. The results show that total 5-year direct costs of reversible contraception management in Spain amount to €12.5 billion. More than half of the cost is attributable to UP, mainly explained by inconsistent or incorrect use of the contraceptive method and a lack of user compliance.^16^

Short-term methods, SARC, are chosen by 87.3% of women using reversible contraception.^3^ Male condom and COC are the methods preferred by young women, based on previous studies published in Spain.^3^,^17^ However, these two methods have the highest failure rates, 18.0% and 9.0% for condom and COC, respectively.^1^,^12^ Women in the middle age range prefer both short- and long-term methods: oral contraception, condom, copper IUD, and LNG-IUS20. The overall LARC use estimated in this study according to Spanish data^3^ is relatively low (12.7% of women using reversible methods), which is consistent with previous estimations about the low use of LARC among adolescents (2.5%) and young adult women (5.4%) in the United States^18^ and by 10.0% of women of childbearing age in Europe, with a mean age of LARC users above 30 years for 57–91% of cases.^19^ The present study shows that long-term methods, such as LNG-IUS20 and copper IUD, are selected by women over 35 years of age who seek safety and user-independent compliance. These methods with lack of dependence on the user’s compliance could reduce the number of UP among young women worldwide, as well as the high economic burden incurred by national health systems, as recognized by world health organizations.^20^,^21^ Implant and LNG-IUS are associated with the lowest failure rates (0.1% and 0.2%, respectively),^1^,^12^ compared with the high rates of SARC methods (18.0% for condom and 9.0% for oral contraceptives, vaginal ring and patch).^1^,^12^ Vaginal ring and patch result in the highest cost per woman in a period of 5 years for the SNHS, while condom and COC also result in higher costs than LNG-IUS and copper IUD. These findings are in line with other published international studies, demonstrating that LNG-IUS, intrauterine devices and implants are the most effective contraceptive methods.^16^

Poor compliance with SARC options may explain the paradoxical association between an increase in elective abortions and an increase in the use of contraception in Spain.^2^ The high rates of UP in Spain might be related to the high use of SARC which, to be effective, need to be associated with user compliance. National surveys show a substantial rate of non-compliance affecting oral contraception,^22^ and inconsistent use of condoms (20.0% of users), particularly by young people.^23^ These two contraceptive methods are the most commonly used in Spain.^3^ Absence of contraception, contraception withdrawal and poor user-compliance are well-known causes of UP resulting in voluntary abortions.^24^–^26^ For example, in a recent study in Spanish women who voluntarily interrupted pregnancy, non-compliant behavior was reported by 77.0% of those using condoms and by 84.0% of those using COC.^5^ Among women employing the vaginal ring, 25.0% reported delay in insertion and among users of the skin patch, 58.0% mentioned delayed application of the patch.^5^

These data reflect the incorrect and inconsistent use of contraception in Spain, resulting in a major public health issue with significant economic and social implications. Based on the data included in the economic analysis, this study demonstrates that LNG-IUS methods and intrauterine devices are the most economic contraceptive methods in Spain in the long term, both for women and the SNHS.

This study has limitations inherent to the analysis. The study was conducted from the perspective of the SNHS and these results cannot be extrapolated to other countries. Public prices of contraceptive methods were calculated according to the catalogues of 3 out of 17 Spanish Autonomous Communities (Andalusia, Catalonia and Madrid). However, these regions are the most populated, representing 48% of the Spanish population. As no Spanish data were available for contraceptive failure rates for ‘typical use’, estimates were obtained from US data.^12^ Additionally, the economic analysis only reflects the most representative healthcare costs associated with the current contraception care pathway in Spain. Adverse events costs related with each contraceptive option (ie, bleeding irregularities, uterine perforation, pelvic infection or ovarian cysts) are not included in the analysis. Further analyses including additional costs, such as adverse events treatment or different contraception therapy failure rates, could yield different results. Despite the study limitations, this analysis could estimate the potentially avoidable costs for the Spanish NHS and provide some guidance as to the benefits of contraception policy implementation.

## Conclusions

Patterns of the use of contraceptives in Spain are characterized by a high use of SARC methods, especially in young women. Spanish women over 35 years of age are more worried about effectiveness and user-independent-compliance, preferring LARC, especially LNG-IUS and copper IUD, as their contraceptive methods of choice. Moreover, these methods show lower 5-year costs related to contraception management and lower withdrawal rates, compared to SARC, resulting in the most economic contraceptive options for women and for the Spanish public health system. Increased use of LARC could contribute to more effective contraception with a significant reduction in UP and potential cost-savings for the SNHS.

## Figures and Tables

**Table 1:** Total Cost of the First Consultation with a Gynecologist to Initiate Contraceptive Therapy

Contraceptive method	Number of women	% of users by method	Average cost per woman of the initial visit (€)*	Total cost associated with initial visits (€ MM)
COC	2 362 775	34.9	79.1	186.9
Gestagen-only OCs	21 777	0.3	79.1	1.7
Vaginal ring	370 204	5.5	79.1	29.3
Patch	54 442	0.8	79.1	4.3
Implant	87 107	1.3	79.1	6.9
Injection	21 777	0.3	79.1	1.7
LNG-IUS12	28 038	0.4	79.1	2.2
LNG-IUS20	351 694	5.2	79.1	27.8
Copper IUD	371 565	5.5	79.1	29.4
Male condom	3 092 295	45.7	79.1	244.6
**Total**	6 761 674			534.8

€ MM: € million.

* Unit costs were calculated according to the average price of the catalogues of public prices of healthcare services of the main Spanish Autonomous Communities (Andalusia,6 Catalonia,^7^ and Madrid^8^) weighted by population and updated to the year 2017, based on the Consumer Price Index.^9^

**Table 2:** Total Cost of Diagnostic Tests to Initiate Contraceptive Therapy

Contraceptive method	Number of women	Ultrasound		Blood test		Average cost per woman (€)	Total cost of diagnostic tests (€ MM)
		Frequency (%)	Cost (€ MM)*	Frequency (%)	Cost (€ MM)*		
COC	2 362 775	70	38.9	30	21.2	25.4	60.1
Gestagen-only OCs	21 777	70	0.4	30	0.2	25.4	0.6
Vaginal ring	370 204	70	6.1	30	3.3	25.4	9.4
Patch	54 442	70	0.9	30	0.5	25.4	1.4
Implant	87 107	70	1.4	30	0.8	25.4	2.2
Injection	21 777	85	0.4	30	0.2	29.0	0.6
LNG-IUS12	28 038	85	0.6	30	0.3	29.0	0.8
LNG-IUS20	351 694	85	7.0	30	3.2	29.0	10.2
Copper IUD	371 565	85	7.4	30	3.3	29.0	10.8
Male condom	3 092 295	0	0.0	0	0.0	0.0	0.0
Total	6 761 674		63.1		32.9		96.0

€ MM: € million.

* Unit costs were calculated according to the average price of the catalogues of public prices of healthcare services of the main Spanish Autonomous Communities (Andalusia,^6^ Catalonia^7^ and Madrid^8^) weighted by population and updated to the year 2017, based on the Consumer Price Index.^9^

**Table 3:** Total Cost of Follow-up Consultations

Contraceptive method	Number of women	Average volume of visits in the first and second year (excluding the initial consultation)	Average volume of visits per year	Average cost of follow-up visits per woman in 5 years (€)	Mean costs for 5-year follow-up visits (€ MM)
COC	2 362 775	2.0	1.0	255.4	603.3
Gestagen-only OCs	21 777	2.7	1.3	347.0	7.6
Vaginal ring	370 204	1.7	0.9	222.6	82.4
Patch	54 442	1.8	0.9	238.3	13.0
Implant	87 107	4.3	2.1	556.5	48.5
Injection	21 777	1.7	0.8	220.0	4.8
LNG-IUS12	28 038	2.1	1.0	271.1	7.6
LNG-IUS20	351 694	3.1	1.6	408.6	143.7
Copper IUD	371 565	3.4	1.7	445.2	165.4
Male condom	3 092 295	1.0	0.5	124.4	384.7
Total	6 761 674				1461.0

€ MM: € million

**Table 4:** Total Cost of Associated Medical Management (initial and follow-up consultations and diagnostic tests)

Contraceptive method	Number of women	Initial visit cost (€ MM)	Mean costs for 5-year follow-up visits (€ MM)	Total cost of diagnostic tests (€ MM)	Total 5-year cost of medical care (visits and diagnostic tests) (€ MM)
COC	2 362 775	186.9	603.3	60.1	850.3
Gestagen-only OCs	21 777	1.7	7.6	0.6	9.8
Vaginal ring	370 204	29.3	82.4	9.4	121.1
Patch	54 442	4.3	13.0	1.4	18.7
Implant	87 107	6.9	48.5	2.2	57.6
Injection	21 777	1.7	4.8	0.6	7.1
LNG-IUS12	28 038	2.2	7.6	0.8	10.6
LNG-IUS20	351 694	27.8	143.7	10.2	181.7
Copper IUD	371 565	29.4	165.4	10.8	205.6
Male condom	3 092 295	244.6	384.7	0.0	629.3
Total	6 761 674	534.8	1461.0	96.0	2091.8

€ MM: € million

**Table 5:** Total Cost of Contraceptive Method Acquisition for a 5-year Period

Contraceptive method	Number of women	Acquisition cost (Retail Price, incl. VAT) (€)*	Cost for the NHS (Retail Price, incl. VAT) (€)*	Frequency	5-year acquisition cost (€ MM)^
COC	2 362 775	11.2	NA (not reimbursed)	Monthly	1723.2
Gestagen-only OCs	21 777	3.4	2.0	Monthly	4.8
Vaginal ring	370 204	19.9	NA (not reimbursed)	Monthly	479.3
Patch	54 442	19.3	NA (not reimbursed)	Monthly	68.3
Implant	87 107	137.5	82.5	3 years	24.0
Injection	21 777	2.1	1.9	3 months	0.9
LNG-IUS12	28 038	153.4	NA (not reimbursed)	3 years	8.6
LNG-IUS20	351 694	95.9”	95.9	5 years	33.7
Copper IUD	371 565	33.3	NA (not reimbursed)	5 years	12.4
Male condom	3 092 295	0.6	NA (not reimbursed)	83/year	757.1
Total	6 761 674				3112.3

€ MM: € million ”Selling price

* Obtained from the SEC survey3 and Bot Plus10

^ Cost associated with the acquisition of each contraceptive method over a period of 5 years

**Table 6:** Total Cost of Unintended Pregnancies (contraception failure)

Contraceptive method	Failure %*	Number of failures in 5 years	Cost of UP in 5 years (€ MM)	Cost per woman of UP in 5 years (€)
COC	9.0%	1 063 249	1904.1	805.9
Gestagen-only OCs	9.0%	9800	17.5	805.9
Vaginal ring	9.0%	166 592	298.3	805.9
Patch	9.0%	24 499	43.9	805.9
Implant	0.1%	218	0.4	4.5
Injection	6.0%	6533	11.7	537.3
LNG-IUS12	0.2%	280	0.5	17.9
LNG-IUS20	0.2%	3517	6.3	17.9
Copper IUD	0.8%	14 863	26.6	71.6
Male condom	18.0%	2 783 066	4984.1	1611.8
Total		4 072 616	7293.5	1078.6

€ MM: € million. UP: unintended pregnancies

* Obtained from 1 and 12

**Table 7:** Total Cost Associated to Reversible Contraception in a Period of 5 Years

Contraceptive method	Cost related to 5-year consultations		Total cost of diagnostic tests		5-year acquisition cost		Cost of UP in 5 years		TOTAL COST in 5 years	
	€ MM	%	€ MM	%	€ MM	%	€ MM	%	€ MM	% Total cost
COC	790.2	17.6%	60.1	1.3%	1723.2	38.5%	1904.1	42.5%	4477.6	35.8%
Gestagen-only OCs	9.3	28.8%	0.6	1.7%	4.8	15.0%	17.5	54.5%	32.2	0.3%
Vaginal ring	111.7	12.4%	9.4	1.0%	479.3	53.3%	298.3	33.2%	898.8	7.2%
Patch	17.3	13.2%	1.4	1.1%	68.3	52.2%	43.9	33.5%	130.8	1.0%
Implant	55.4	67.6%	2.2	2.7%	24.0	29.2%	0.4	0.5%	81.9	0.7%
Injection	6.5	32.9%	0.6	3.2%	0.9	4.7%	11.7	59.2%	19.8	0.2%
LNG-IUS12	9.8	49.8%	0.8	4.1%	8.6	43.6%	0.5	2.5%	19.7	0.2%
LNG-IUS20	171.5	77.4%	10.2	4.6%	33.7	15.2%	6.3	2.8%	221.7	1.8%
Copper IUD	194.8	79.7%	10.8	4.4%	12.4	5.1%	26.6	10.9%	244.6	2.0%
Male condom	629.3	9.9%	0.0	0.0%	757.1	11.9%	4984.1	78.2%	6370.5	51.0%
Total	1995.7	16.0%	96.0	0.8%	3112.3	24.9%	7293.5	58.4%	12 497.6	100%

€ MM: € million

% Total cost: contribution of the cost of each method to the overall 5-year cost.

## Abbreviations

AE: Adverse Events
COC: Combined Oral Contraceptives
IUD: Intrauterine Device
LARC: Long-Acting Reversible Contraceptives
LNG-IUS: Levonorgestrel-releasing intrauterine system
LNG-IUS12: Levonorgestrel-releasing intrauterine system Jaydess 12μg/day
LNG-IUS20: Levonorgestrel-releasing intrauterine system Mirena 20μg/day
MHSSE: Ministry of Health, Social Services and Equality
OCs: Oral Contraceptives
SARC: Short-Acting Reversible Contraceptives
SEC: Spanish Society of Contraception
SNHS: Spanish National Health System
UP: Unintended Pregnancies
